# Occupational Benzene Exposure and Lymphoma Risks

**DOI:** 10.1289/ehp.1104167

**Published:** 2011-11-01

**Authors:** Tom Sorahan

**Affiliations:** Institute of Occupational and Environmental Medicine, University of Birmingham, Edgbaston, Birmingham, UK, E-mail: T.M.Sorahan@bham.ac.uk

In their recent meta-analysis, Vlaanderen et al. (2011) claimed to show evidence for associations between occupational benzene exposure and risks of multiple myeloma, acute lymphocytic leukemia, and chronic lymphocytic leukemia. However, one of the larger available studies, including 5,514 benzene-exposed UK workers (Sorahan et al. 2005), was excluded from this meta-analysis, apparently because the study had an elevated standardized mortality ratio (SMR) for secondary and unspecified cancers. On the basis of national mortality rates, we would have expected 7% of all cancer deaths in the UK study to have been in the unspecified category (e.g., carcinomatosis, mesothelioma with site unspecified); however, 9% of deaths were unspecified. Given the size of the study (2,430 deaths from all causes), this difference was statistically significant (Sorahan et al. 2005). Is it reasonable to conclude that a study with 93% of cancer deaths with site of cancer specified is informative but one with only 91% specified is not? I do not believe that it is. Vlaanderen et al. (2011) are of course free to come to a different conclusion, but any conclusion they reach must be implemented in an even-handed way. Some obvious questions then arise: *a*) How elevated did the SMR for unspecified cancers have to be for a study to be excluded from their meta-analysis? *b*) Were all the other studies assessed against this criterion? *c*) How many studies did not provide enough information for this criterion to be assessed? *d*) Why was this number not supplied by Vlaanderen et al. (2011)?
